# Data on circulating leukocyte subpopulations and inflammatory proteins in children with familial hypercholesterolemia and healthy children

**DOI:** 10.1016/j.dib.2016.12.042

**Published:** 2016-12-29

**Authors:** Jacob J. Christensen, Liv Osnes, Bente Halvorsen, Kjetil Retterstøl, Martin P. Bogsrud, Cecilie Wium, Arne Svilaas, Ingunn Narverud, Stine M. Ulven, Pål Aukrust, Kirsten B. Holven

**Affiliations:** a Department of Nutrition, Institute of Basic Medical Sciences, University of Oslo, P.O box 1046 Blindern, Oslo 0317, Norway; b The Lipid Clinic, Oslo University Hospital Rikshospitalet, P.O box 4950 Nydalen, Oslo 0424, Norway; c Department of Immunology, Oslo University Hospital Rikshospitalet, P.O box 4950 Nydalen, Oslo 0424, Norway; d Research Institute of Internal Medicine, Oslo University Hospital Rikshospitalet, P.O box 4950 Nydalen, Oslo 0424, Norway; e Institute of Clinical Medicine, University of Oslo, P.O box 1171 Blindern, Oslo 0318, Norway; f K.G. Jebsen Inflammatory Research Center, University of Oslo, P.O box 1171 Blindern, Oslo 0318, Norway; g Norwegian National Advisory Unit on Familial Hypercholesterolemia, Oslo University Hospital Rikshospitalet, P.O box 4950 Nydalen, Oslo 0424, Norway; h Section of Clinical Immunology and Infectious Diseases, Oslo University Hospital Rikshospitalet, P.O box 4950 Nydalen, Oslo 0424, Norway

## Abstract

The data in this relies on a previous publication: “Altered leukocyte distribution under hypercholesterolemia: a cross-sectional study in children with familial hypercholesterolemia” (Christensen et al. 2016) [1]. In the present paper, whole blood leukocyte distribution and plasma inflammatory proteins were measured for association with cholesterol concentration and CRP in children with familial hypercholesterolemia (FH) and healthy children.

**Specifications Table**

**Table:** 

Subject area	*Biology*
More specific subject area	*Circulating leukocytes and plasma inflammatory proteins*
Type of data	*Tables, figures*
How data was acquired	*Gallios Flowcytometer (Beckman Coulter, San Diego, CA) or a Canto II Flowcytometer (BD), and enzyme immunoassays from R&D Systems (Minneapolis, MN)*
Data format	*Analyzed*
Experimental factors	*Whole blood leukocyte distribution and plasma level of inflammatory proteins*
Experimental features	*Correlation between monocytes and lipids or CRP; correlation between monocytes and lymphocytes; distribution of leukocytes; and concentration of inflammatory proteins in familial hypercholesterolemia (FH) and healthy children*
Data source location	*The Lipid Clinic, Oslo University Hospital Rikshospitalet, and Department of Nutrition, University of Oslo, Oslo, Norway*
Data accessibility	*The data are within this article*

**Value of the data**

• Circulating leukocyte subpopulations and inflammatory proteins could be markers of atherosclerosis development in children with familial hypercholesterolemia (FH).
• The data are useful for scientists interested in markers of development of atherosclerosis in relation to high cholesterol in childhood.
• The data may provide insight on how hypercholesterolemia and inflammation are interconnected in the early phase of atherosclerotic development.
• The method can be used for further investigation of cellular or soluble markers of atherosclerosis development in relation to high cholesterol in childhood.

## 1 Data

**Fig. 1:**
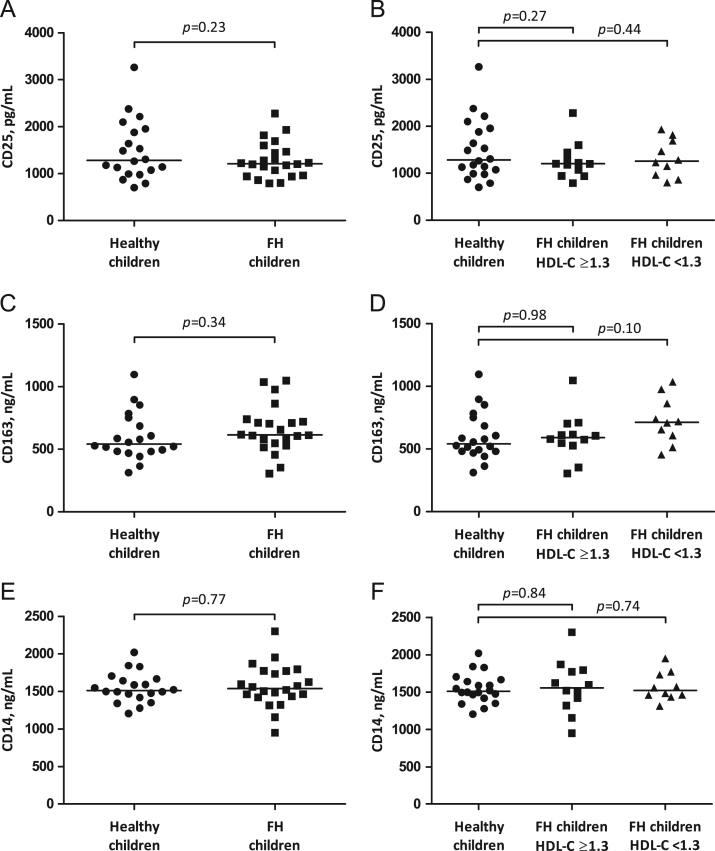
Serum concentration of lymphocyte and monocyte markers. The panels display cluster of differentiation (CD) 25 (A, B), CD163 (C, D) and CD14 (E, F), as determined by enzyme immunoassays. Lines represent median values within each group. *p* values are independent samples *t*-test using non-transformed data.

**Fig. 2:**
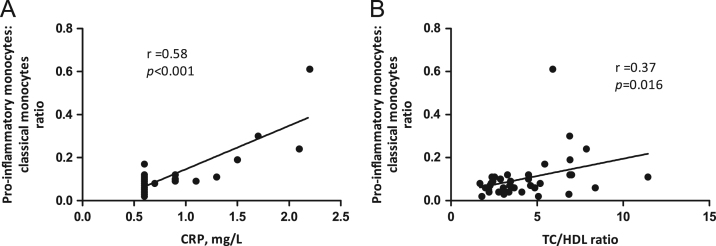
Correlation between pro-inflammatory monocytes and markers of cardiovascular risk. The panels display correlation between pro-inflammatory: classical monocytes ratio and CRP (A) and TC/HDL ratio (B). *r* is Spearman׳s rho (non-parametric) correlation coefficient. *n*=41–43. CRP, C reactive protein; HDL-C, HDL cholesterol; TC, total cholesterol.

**Fig. 3:**
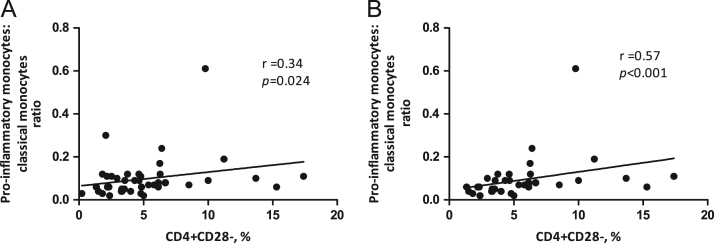
Correlation between pro-inflammatory monocytes and pro-inflammatory lymphocytes. The panels display correlation between pro-inflammatory:classical monocytes ratio and pro-inflammatory CD4+CD28- lymphocytes in all children (A, *n*=43) and children below 13 years of age (B, *n*=36). *r* is Spearman׳s rho (non-parametric) correlation coefficient.

Whole blood leukocyte distribution and plasma level of inflammatory proteins were evaluated for association with cholesterol level and CRP. Data includes correlations between leukocyte subpopulations, cholesterol and CRP in FH children and healthy children combined (Table 1, and Fig. 2, Fig. 3), and comparison of leukocyte distribution in FH children and healthy children less than 13 years of age (Table 2 and Fig. 1).

## 2 Experimental design, materials and methods

Data are from cross-sectional study in FH children and healthy children, thoroughly explained elsewhere [1]. Briefly, we recruited children with a definite diagnosis of heterozygous FH from the Lipid Clinic, Oslo University Hospital Rikshospitalet, Oslo, Norway. Control children without FH, herein referred to as “Healthy children”, were recruited in the same time period. For all children, we collected the following: 4 mL heparin plasma for measurement of CRP and lipids, 4 mL EDTA whole blood for characterization of leukocyte subpopulations, and 5 mL serum for analysis of inflammation markers. CRP and lipids were measured using highly standardized protocols at the Department of Medical Biochemistry at Rikshospitalet [1], Oslo, whereas B- and T-cell subpopulations and monocyte subpopulations were analyzed by flow cytometry at the Department of Immunology at Rikshospitalet, Oslo, as described previously [1]. For CRP, lipids and flow cytometry, analyses were performed on the same day as sampling. Serum samples were processed and stored at −80 °C until study completion, followed by measurements of concentration of cluster of differentiation (CD) 163, CD14 and CD25 using enzyme immunoassays from R&D Systems (Minneapolis, MN).

Statistical analyses were performed similarly as in [1]. Briefly, the data are presented as mean (standard deviation) or median (25th–75th percentile). Whereas independent samples *t*-test was used for parametric data, we log-transformed the variables and used independent samples *t*-test for non-parametric data. Because of skewed distributions, we report Spearman׳s rank correlation coefficient in the correlation analyses. Alpha level of significance was set to 5%, and SPSS (v22.0, IBM) was used for all calculations.

## Figures and Tables

**Table 1:** Correlations between monocyte subpopulations and HDL-C, LDL-C, and CRP concentration.

	CRP, mg/L		LDL-C, mmol/L		HDL-C, mmol/L		TC/HDL ratio	
	*r*	*p*	*r*	*p*	*r*	*p*	*r*	*p*
***Monocytes:***								
Classical monocytes, %	**−0.56**	**<0.001**	−0.21	0.18	0.20	0.22	−0.23	0.15
Pro-inflammatory monocytes, %	**0.47**	**0.002**	0.26	0.096	−0.29	0.070	0.29	0.065
Non-classical monocytes, %	0.26	0.10	0.14	0.40	0.04	0.80	0.07	0.65
Pro-inflammatory:classical monocytes ratio	**0.58**	**<0.001**	0.31	0.050	**−0.36**	**0.022**	**0.37**	**0.016**

Data are Spearman׳s rho (non-parametric) correlation coefficient. *n*=41–43. CRP, C reactive protein; HDL-C, HDL cholesterol; LDL-C, LDL cholesterol; TC, total cholesterol.

**Table 2:** Whole blood leukocyte populations characterized by flow cytometry (under the age of 13).

	FH children	Healthy children	
***Monocyte subsets:***			*p*^a^
Classical monocytes (%)	86.1 (77.6–89.8)	87.9 (84.3–89.9)	*0.094*
Pro-inflammatory monocytes (%)	6.5 (4.8–10.9)	5.4 (3.7–7.8)	0.21
Non-classical monocytes (%)	6.7 (3.8–8.5)	6 (3.5–8.1)	0.13
Pro-inflammatory:classical monocytes ratio	0.08 (0.06–0.14)	0.06 (0.04–0.09)	***0.037***
CD18, MFI	15.3 (6.9–37.4)	10.1 (6.0–24.8)	0.29
CD11b, MFI	15.6 (11.5–29.7)	13.3 (10.8–26.9)	0.57
CD31, MFI	19.3 (18.5–21.5)	19.5 (17.7–22.5)	0.51
***Distribution of lymphocytes:***			
T cells (CD3+), cells/µL	1664.6 (577.7)	1901.8 (476.9)	0.21
T helper cells (CD4+), cells/µL	921.8 (344.6)	1047.7 (254.2)	0.24
Cytotoxic T cells (CD8+), cells/µL	630 (288)	732.5 (254.9)	0.29
NK cells (CD16+), cells/µL	241.8 (84)	281.7 (201.7)	0.46
B cells (CD19+), cells/µL	388.7 (229.2)	438.2 (177.2)	0.49
T cells (CD3+) (%)	72.2 (6.5)	72.9 (7.3)	0.77
T helper cells (CD4+) (%)	39.5 (8.1)	40.2 (7.9)	0.78
Cytotoxic T cells (CD8+) (%)	27.1 (5.1)	27.7 (6)	0.78
NK cells (CD16+) (%)	10 (8–13.5)	9 (6.5–14.5)	0.71
B cells (CD19+) (%)	16.6 (5.1)	16.8 (5.3)	0.90
***CD4 T cell subsets:***			
Recent thymic emigrant (RTE) (%)	70.9 (11.2)	78.7 (9.3)	***0.028***
Naive CD4+ cells (%)	76.4 (11.3)	70.1 (10.9)	*0.098*
Naive CD4+: RTE cells ratio	1.1 (0.9–1.3)	0.9 (0.8–1)	***0.014***
Memory cells (%)	41.5 (11.6)	38.7 (11.8)	0.48
Follicular cells (%)	6 (4.1–7.1)	6.4 (5.4–7.8)	0.28
T regulatory cells %	5.4 (1.2)	5.3 (1.2)	0.95
Cytotoxic cells (CD28−) (%)	6.2 (3.7–8.8)	4.2 (2.8–6.3)	*0.079*
Activated T cells (CD25+) (%)	15.3 (5.8)	14.7 (4.9)	0.74
***CD8 T cells:***			
Naive CD8+ cells, %	66.2 (18.8)	70 (12.1)	0.48
Early effector memory cells, %	13.5 (9.1–22.8)	12 (9.6–15.8)	0.71
Late effector memory cells, %	13.2 (1.8–22.7)	12.3 (5.8–24.7)	0.48
***B cells:***			
Naive B cells, %	79.6 (70.3–84.3)	74.9 (71.3–83.4)	0.34
IgM memory cells, %	9.3 (4.3)	7.9 (3.1)	0.27
Class switched cells, %	5 (4.1–9)	7.3 (5.2–8.3)	0.25
Transitory cells, %	5.4 (4.2)	5.4 (3.1)	0.96
Plasmablast cells, %	0.6 (0.2–0.9)	0.4 (0.2–1.2)	0.82
CD21low cells, %	1.8 (1.2–3.2)	2.4 (1.5–2.9)	0.55

Data are presented as mean (SD) or median (25th–75th percentile). *n*=16–18 for FH children; *n*=17–18 for healthy children. CD, cluster of differentiation; MFI, median fluorescence intensity; NK, Natural killer; RTE, Recent thymic emigrant.

a Independent samples *t*-test (non-transformed data) for parametric data, and independent samples *t*-test (log-transformed data) for non-parametric data. Italic font indicates *p* value less than 0.10, and italic and bold font indicates *p* value less than 0.05.
