# Supplementary material for: Data on circulating leukocyte subpopulations and inflammatory proteins in children with familial hypercholesterolemia and healthy children
Source: Data Brief. 2016 Dec 29;10:587–92. doi: 10.1016/j.dib.2016.12.042 (PMC5219596; doi:10.1016/j.dib.2016.12.042)
Supplement: Supplementary file 1 — Supplementary material [file mmc1.docx]

**Conflict of interest**

Dr. Bogsrud has received research grants and/or personal fees from Amgen, Sanofi, MSD, Aegerion, none of which are related to the contents of this manuscript. Dr. Retterstøl has received research grants and/or personal fees from Merck, Chiesi, Takeda, The Directorate for Health, Norway, The Norwegian Medical Association, Mills, none of which are related to the contents of this manuscript. Dr. Ulven has received grants from Mills DA, TINE DA, and Olympic Seafood, none of which are related to the contents of this manuscript. Dr Holven has received research grants and/or personal fees from Tine DA, Mills DA, Olympic Seafood, Amgen, Sanofi and Pronova; none of which are related to the contents of this manuscript. The other authors have no financial relationships relevant to disclose.

We confirm that the manuscript has been read and approved by all named authors and that there are no other persons who satisfied the criteria for authorship but are not listed.
